# Variability of Chemical Profile in Almonds (*Prunus dulcis*) of Different Cultivars and Origins

**DOI:** 10.3390/foods10010153

**Published:** 2021-01-13

**Authors:** Ana Beltrán Sanahuja, Salvador E. Maestre Pérez, Nuria Grané Teruel, Arantzazu Valdés García, María Soledad Prats Moya

**Affiliations:** Analytical Chemistry, Nutrition and Food Sciences Department, University of Alicante, P.O. Box 99, E-03080 Alicante, Spain; salvador.maestre@ua.es (S.E.M.P.); nuria.grane@ua.es (N.G.T.); arancha.valdes@ua.es (A.V.G.); maria.prats@ua.es (M.S.P.M.)

**Keywords:** almond (*Prunus dulcis*), cultivar, geographical authenticity, chemical profile, analytical techniques, multivariate analysis, classification

## Abstract

Almonds show a great variability in their chemical composition. This variability is a result of the existence of a diverse range of almond cultivars, the self-incompatibility of most almond cultivars, and the heterogeneous harvesting conditions found around the different locations where almons are grown. In the last years, the discrimination among almond cultivars has been the focal point of some research studies to avoid fraud in protected geographical indications in almond products and also for selecting the best cultivars for a specific food application or the most interesting ones from a nutritional point of view. In this work, a revision of the recent research works related to the chemical characterization and classification of almond cultivars from different geographical origins has been carried out. The content of macronutrients, tocopherols, phytosterols, polyphenols, minerals, amino acids, and volatile compounds together with DNA fingerprint have been reported as possible cultivar and origin markers. The analysis of the results showed that no individual almond compound could be considered a universal biomarker to find differences among different almond cultivars. Hence, an adequate selection of variables or the employment of metabolomics and the application of multivariate statistical techniques is necessary when classification studies are carried out to obtain valuable results. Meanwhile, DNA fingerprinting is the perfect tool for compared cultivars based on their genetic origin.

## 1. Introduction

**Figure 1:**
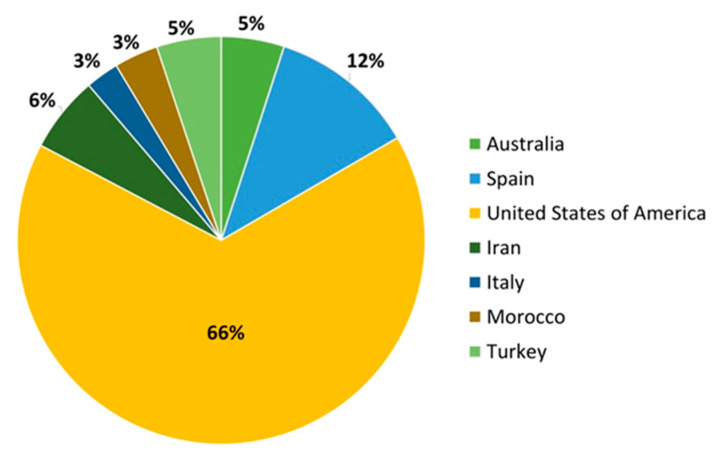
Worldwide almond production (2019) [2].

According to the International Nuts & Dried Fruits Statistical Yearbook 2018/2019, almonds are the most consumed nut in high-income economies, accounting for 39% and followed by walnuts, cashews and hazelnuts [1]. Nowadays, the USA is the leading producer of almonds, representing nearly 66% of the worldwide production, followed by Spain (Figure 1) [2]. The worldwide production of almonds has been noticeable increased in the last years (Table 1). USA production is centred in California, being Non-Pareil the first in the top ten produced almond cultivars [3]. Meanwhile, in Europe, almond production is carried out under different climate conditions being the production conditioned by this fact. Due to the wide diversity in climate of Spain, almond producers have selected genotypes to avoid decreases in production. In this sense, the Agrifood Research and Technology Centre (CITA) maintains the National Almond Collection that contains most of the Spanish almond cultivars.

**Figure 2:**
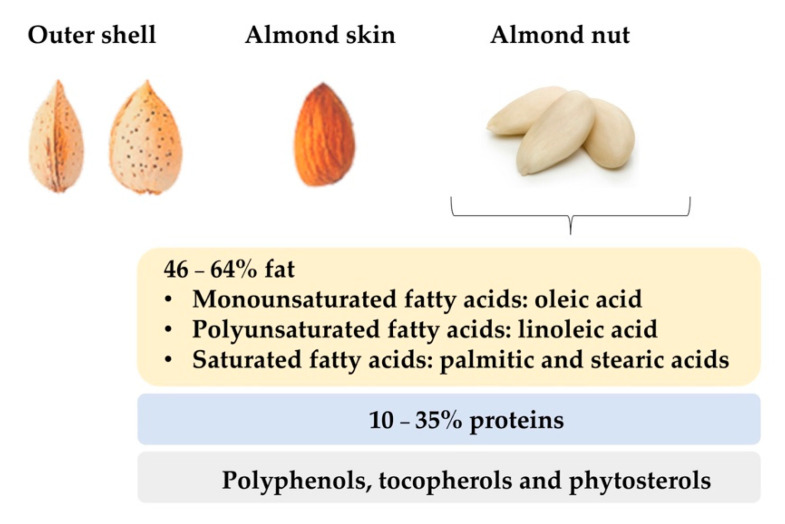
Physical and nutritional characteristics of almond nut.

Almond (*Prunus dulcis*) is a tree species that together with peach is included in the subgenus *Amygdalus* [4]. Sweet almonds have an average length of 2.3 cm, 1.4 cm width, and 0.8–1.0 cm thickness. They have a delicate, aromatic and sweet flavour. Externally, the seeds are oval, asymmetrical, flattened, sharpened at one end and rounded at the other, with an external husk that protects them from the environmental conditions and harvest. Finally, the almond nut is surrounded by the almond skin, also called tegument, which is a thick and brown wrapper, somewhat rough with quite noticeable streaks (Figure 2). Almond kernels have low water content, while its amount of fat can range from 46% to 64% [5] and their protein level can be around 10–35% [4]. It is known that these values are conditioned by cultivar and geographical origin [6]. Regarding the composition of the fat fraction, almonds are characterized by high amounts of monounsaturated and polyunsaturated fatty acids [7]. Oleic and linoleic acids are the most abundant unsaturated fatty acids in almonds, accounting for about 80–90% while saturated fatty acids, such as palmitic and stearic fatty acids, are present in lower quantities (<10%) [8]. Almonds are also noticeable for their content in minor compounds such as polyphenols, and tocopherols [9], which are correlated with antioxidant properties that reduce the risk of suffering from diseases related to oxidative stress such as arthritis, vasculitis, and high blood pressure, cancer or Alzheimer [10]. Other minor compounds present in almonds are phytosterols or plant sterols which carry out cell functions in the plants analogous to those of cholesterol in animals.

The commercial interest of almonds is increasing because they greatly enrich many recipes and desserts in Mediterranean cuisine such as nougat, marzipan, pralines or ice creams. Also, they can be used in different forms: natural or salty, fresh or dried, roasted or fried [11]. Several publications concluded that different almond cultivars showed dissimilar chemical composition values and physicochemical and biochemical properties [12,13]. Due to this fact, the discrimination among almond cultivars has been the focal point of many research studies in order to avoid frauds in the food industry. Thus, it is interesting to check and evaluate the research works related to the chemical characterization and classification of almond cultivars. This review aims to discuss and summarize all recent studies related to the selection of chemical markers that have been employed to identify the botanical and/or the geographical origin in almonds.

## 2. Total Fat, Fatty Acids and Triacylglycerides

The USDA National Nutrient Database highlighted the value of 49.9 g total lipid per 100 g raw almonds as a Standard Reference value [14], but the composition depends not only on cultivar but also on growing conditions and year of cultivation, among others. Related to this fact, in a study carried out in our research group, values of lipid content between 42.5–52.0%, 50.9–60.9%, 46.6–56.2%, and 47.9–56.2% were reported for the cultivars Garrigues, Marcona, Guara, and Butte, respectively. All of them were grown in different locations of Spain and California. Samples from two consecutive years were analyzed, confirming that the oil content is dependent on the origin and year of cultivation [9].

The fat fraction of almonds is mainly composed of fatty acids with 14 carbons up to 20 carbons quantified mainly by gas chromatography-flame ionization detector (GC-FID) [15]. In general terms, the amount of unsaturated fatty acids account for more than 90% of total lipids, being the main ones in decreasing order of mention: oleic (58.1–71.3%), linoleic (15.7–29.9%), palmitoleic (0.20–0.62%), and vaccenic acid (0.77–2.17%). In contrast, saturated fatty acids, such as palmitic (5.9–7.5%), stearic (1.0–2.4%), arachidic (0.07–0.10%), and myristic (0.02–0.05%) represented only the 10% of total lipids [6]. Different research works in which almonds from different parts of the world were analyzed (i.e., almonds from Turkey [16], Spain [15], Italy [17], Serbia [18], China [19], and California [15], among others) reported oleic and linoleic as the major fatty acids.

In a recent study, it was revealed that the quality of the almond kernel depends on the maturity stage of the fruit, being noticeable the fat fraction changes related to the fatty acid composition [20]. Moreover, other studies have suggested that poor water supply to the crop leads to a lower oleic/linoleic ratio indicating a significant effect of irrigation on almond fatty acid composition [21]. In this sense, the irrigation management and the temperatures were the main factors affecting the oil content and fatty acid composition studied in seventeen different almond cultivars grown in two different environmental conditions, such as northeast Spain and central Morocco [22]. Regarding samples grown in Spain submitted to lower temperatures and better water contribution, the values of total oil content (58.65% vs. 55.58% (*w*/*w*)) and the percentage of oleic acid (71.1% vs. 68.6% (*w*/*w*)) were higher in comparison with the ones obtained in samples grown in central Morocco.

Considering the genetic diversity, the fatty acid composition has been strongly influenced by the genotype [22] being the oleic and linoleic the most variable acids among genotypes [23]. Kodad et al. studied samples of forty-seven advanced self-compatible almond genotypes in terms of the analysis of oil content and fatty acid composition [8]. The analyzed samples were grown in two different years and belonged to eight cultivars developed in an almond reproducing program. The obtained results confirmed that these parameters were highly variable, being strongly influenced by genotype.

Crucial aspects of food safety are the food authenticity studies focused on the identification of the geographical origin of food samples. In these studies, it is essential the development of new analytical methods and techniques able to confirm the chemical composition detailed on the food label [17]. Following this work line, a recent study reported a proper classification of almonds from different geographical origins (Sicily, Spain and California) by the combination of chemometric techniques and the data related to fatty acid composition achieving an 87% of correctly classified samples. In this way, linear discriminant analysis (LDA) is the most predominant chemometric technique used with this purpose among the supervised pattern recognition methods [17]. Also, Colic et al. [18] determined the total oil, fatty acids total phenolic content and the radical-scavenging activity in almonds belonging to North Serbia from cultivars Marcona, Texas, and Troito. Regarding the fatty acid composition, oleic and linoleic acids were the most abundant ones among the sixteen compounds that were identified. To find out the components able to differentiate among samples based on their genotype, principal component analysis (PCA) was performed, with principal component 1 being strongly influenced by oleic, pentadecanoic, and palmitoleic acids content.

Other researchers analyzed the fatty acid composition of different almond cultivars grown in Afghanistan and determined the levels of palmitic, palmitoleic, tridecanoic, stearic, oleic, linoleic, arachidic, linolenic, henicosanoic, behenic, tricosanoic, and lignoceric acids [24]. Taking into account all the determined fatty acids, significant differences (*p* < 0.05) between the almond cultivars were found and the study revealed that the LDA classification was mainly influenced by linolenic, henicosanoic, tridecanoic, tricosanoic, and lignoceric acids. In similar research work, commercial almond samples from different cultivars grown under the same environmental conditions (Cristomorto, D. Largueta, Ferraduel, Ferragnes, Ferrastar, Glorieta, Lauranne, Masbovera, Nonpareil, Picantili, Sonora, Supernova, Texas, Tuono, and Yaltinski) were analyzed by Yildirim et al. [7]. PCA analysis showed that principal component 1 was mainly contributed by palmitic, palmitoleic, stearic, oleic and arachidic acid, unsaturated fatty acids (UFA), saturated fatty acids (SFA) and UFA: SFA ratio. In a similar study, Beltrán et al. [15] achieved a classification of four almond cultivars (Butte from USA, Marcona, Guara and Garrigues from Spain) by using parameters related to the oil degradation. As it was expected, the main fatty acids found in the almond samples were oleic, linoleic, stearic, palmitic and palmitoleic. The content in linoleic acid was higher in samples belonging to Butte cultivar in comparison with the Spanish ones (Marcona, Guara and Garrigues) and the application of LDA technique provided a 100% correctly classification of samples according to the cultivar.

Chemometric characterization of almond germplasm was conducted by Kodad et al. [25]. The oil content and the main fatty acids were determined in 73 almond cultivars from 10 different countries (Spain, Argentina, France, Italy, Greece, India, Syria, Portugal, Ukraine and United States). The application of PCA indicated that the responsible variables for the separation were palmitic, oleic, and linoleic acids and the oleic acid/linoleic acid ratio indicating that fatty acid composition is strongly affected by the cultivar.

On the other hand, almond oil has been reported as the nut oil with the highest content in triacylglycerol′s (TAGs) (about 98%). However, the determination of the almond oil TAGs composition has not been the focus of many scientific studies. Among the few studies found, HPLC with refractive index detection was employed by Prats et al. [26] to quantify the TAGs present in different almond cultivars such as Desmayo Largueta, Marcona, Guara, and Masbovera from Spain; Texas, Non Pareil, and Titan from the United States of America; Tuono from Italy; Ferragnes from France; and Primorskyi from a Caucasian region. The main triacylglycerol detected was OOO followed by OLO, POO, OLL, PLO, StOO, LLL, PLL, and PLP, where O refers to oleic acid, L to linoleic acid, P to palmitic acid, and St to Stearic acid, with OOO and OLO together representing more than 60% of the total triglyceride content. A correct classification was obtained based only on the almond TAGs determined except PLP and POO by using four discriminant functions with the calculated retain variables. The classification was based on almond genotypes which were not camouflaged by environmental conditions.

To look for dissimilarities among Protected Designation of Origin (PDO) Amêndoa Douro and commercial non-PDO cultivars, Barreira et al. [27] characterized almonds during three harvesting years in Portugal in terms of fatty acid profile and TAGs. Accordingly, OOO and OLO were the major TAGs present in the studied samples as it was previously reported by Prats et al. [26]. To obtain statistical differences among PDO and non-PDO cultivars independent of the grown year, the PCA, LDA, and analysis of variance chemometric techniques were applied obtaining good results with the data of TAG analysis coupled with LDA.

From the fatty fraction, it seems that fatty acids and triacylglycerols could be used to classify almond cultivars if a good selection of variables is done when multivariate analysis is applied. The variables that have had more differentiating power in the literature (Table 2) consulted are: palmitic, oleic, and linoleic acids and the oleic acid/linoleic acid ratio together with the following triglycerides OOO, OLO, POO, OLL, StOO, LLL, and PLL.

## 3. Proteins

The levels of protein in almonds can vary from 10% to 35% [4]. Usually, protein concentration is determined from the nitrogen levels using a nitrogen-to-protein conversion factor. In this sense, it is interesting to note that 6.25 or 5.18 values for this factor are reported, which can lead to differences in the stated protein content of almonds. As it has been reported, protein content in almond kernels steadily increases up to harvest, since the seed has a greater protein synthesis activity [30] and, additionally, its water content is reduced. Among the studies found, Barreira et al. evaluated the protein content of nine almond cultivars collected through three consecutive years in Portugal [31]. In this work, the authors used a nitrogen-to-protein conversion factor of 5.18 but non statistically signifficant differences in the protein levels were found. The same factor was used in another study [32] and the authors found that the proteins were one of the major components that had more variability related to the year of cultivation. In contrast, Drogoudi et al. [33], using the same protein conversion factor, showed that proteins have the highest variability between genotypes and the lowest between years, so it could be a good marker to differentiate varieties. Previously, this conclusion had been stated in the work of Calixto et al. [34] which determined the protein content of five different almond cultivars collected during the same year using a conversion factor of 6.25.

In the paper of Rabadán et al. [32], the protein concentration was the nutritional component with the highest reported variability among kernels, with the crop year being responsible for most of this variability. However, Yada et al. [35] found that, although the protein concentration was significantly different for two of the five cultivars studied, the crop year and growing region had not impact on the three genotypes followed. In this line, Kodad et al. [36] selected 41 almond genotypes from four different regions of Morocco during two consecutive years and evaluated the protein content of the kernel using the Dumas method with a conversion factor of 6.25. The ANOVA of the protein levels showed that the effect of genotype was significant as were the year and regions, and the interaction of year and genotype. The PCA carried out showed that kernels oil-to-protein ratio could be used to differentiate genotypes. Finally, other works have also found differences in the protein content among several cultivars, although no classification was attempted [37]. From these results, it appears that the protein content of almond cultivars could hardly, on its own, be a candidate marker for cultivar classification as the influence of the growing conditions and year of cultivation are determinant.

## 4. Amino Acids

Amino acids accumulate in the almond kernel until the protein synthesis activity begins, afterwards, the levels of amino acids stabilize leading to a final residual (i.e., <200 mg/100 g) free amino acid content in the ripe kernel [30]. Font i Forcada et al. [38] reported that the heritability estimate of protein content in almond was very low, confirming the strong effect of environmental conditions on its expression. This was evidenced by the differences in this parameter found when different irrigation systems with inorganic/organic fertilization schemes were used [39]. Some works showed the complete amino acid profile of different almond cultivars [40,41]. However, the studies were not focused on comparison for classification purposes.

Furthermore, some articles of our research group showed data of the free amino acid profile of almond kernels, using this information for cultivar classification purposes [42,43,44]. Seron et al. [44] studied the free amino acid composition of nineteen cultivars from different countries, Spain, USA, Australia, Italy, and Tunisia, belonging to the same crop year. Leucine, Valine, and Alanine were the amino acids with a higher cultivar discrimination power although all amino acids contributed to differentiate cultivars. By applying PCA and discriminant analysis, the Spanish cultivars could be classified as a single class different from the rest. In the work of Grané et al. [42], the free amino acid profile of five almond cultivars grown in different regions of Spain was used as a classification tool. Two groups were found with Serine and Asparagine levels being the more effective data for differentiating cultivars. Finally, in a study using ten different cultivars grown in different parts of Spain, the authors suggested that the cultivar variable had a stronger influence on the free amino acid profile than the variables such as the region of growing and weather conditions [43]. Hence, Asparagine and Glutamic acid levels were used to distinguish Marcona and Texas cultivars from other eight cultivars using LDA.

## 5. Carbohydrates and Dietary Fibre

Carbohydrates are present in almond kernels in the 2–12% range [45], mainly as soluble sugars. Sucrose and raffinose are the main compounds of this group, representing about 90% of the total sugars level when the seeds are ripe [30,31,46]. Meanwhile, dietary fibre levels are around 10% [46] which can be important from a human nutrition perspective.

Sucrose was quantified in seven almond cultivars and the authors found significant differences among cultivars but not between different growing years and different growing regions for the same genotype [35]. In another study, the sugar content of almonds was determined, using the anthrone method, and different concentrations were described among the twelve cultivars analysed, it has to keep in mind that all samples were collected in the areas of Turkey with similar ecological conditions [37], however no conclusions linked to the genotype were drawn from these data. In contrast, in another study [32] the content of carbohydrates was also mainly determined by the crop growing conditions when data from two consecutive years and ten cultivars were combined. It is important to mention that in this report the carbohydrate values were obtained by the difference method which could induce the high variability of the values observed. This conclusion was also reached when new almond varieties were characterized [47]. In a different study [48], the free sugar profile from twelve Tunisian almond cultivars and five almond cultivars from France, Italy and Spain were analyzed by a high-performance liquid chromatography over two years (2009–2010). PCA was performed on biochemical data (fatty acid, total oil and protein contents and sugar composition) for screening and describing the similarities among the 17 studied almond cultivars. From results, authors concluded that PC-1 was mainly contributed by total sugar, sucrose and raffinose contents accounted for 27.41% of the total variance. As observed in this work, PC-1 allowed the separation of some varieties due to their highest content in these parameters, mainly ‘Porto’, ‘Fournat de Breznaud’, ‘Blanco’, ‘Dillou’, ‘Khoukhi’, and ‘Lauranne’ almond cultivars. Results evidenced that sugar contents in almond depend of a polygenic background with a clear environment effect.

Almond fibre is mainly composed of cellulose, hemicelluloses, and lignin, which account up to 80% of the total fibre content of kernels [45]. It is important to note that different methods of determination of fibre content have been used, mainly neutral detergent fibre, acid detergent fibre, crude fibre, and total dietary fibre, which affect the values reported. Soler et al. [30] demonstrated that the levels of neutral detergent fibre increased with fruit development until the synthesis activity of oil used part of these compounds and its accumulation slowed down. This parameter has shown some variability among genotypes, hence in the study carried out with 10 cultivars through three years this variability was mainly attributed to the genotypes studied [32]. However, in other works results showed that fibre contents have a strong environmental influence hence, when the detergent method was employed, the fibre content (i.e., neutral detergent fibre, acid detergent fibre and cellulose) possessed very limited differentiation ability regarding almond cultivar discrimination in a three year to follow up study [27]. A similar conclusion was reached by Yada et al. [35] who was unable to find statistically significant differences among seven cultivars in the three-year study for the contents of total dietary fibre, but they did find differences among the cultivation years. In this study, the AOAC 991.43 method for total dietary fibre determination was employed, this was also confirmed by Romero et al. [47]. As a final remark, it may be concluded that the use of carbohydrate and fibre contents as parameters to differentiate almond varieties have been less exploited than fat content or fatty acids, perhaps due to the lack of a unified method for determination or the influence of agronomical variables.

## 6. Minerals

The almond kernel is considered a good source of minerals [4,45]. The majority of the studies provide data on major elements: K, P, Ca, and Mg (found at levels above few hundreds mg/100 g wet basis), and some minor elements: mainly Na, Fe, Cu, Zn, and Mn (usually in the mg/100 g wet basis level or below) [4,35]. Fewer studies provide data on other minor elements, such as Li, Sr, Al [17], B [40], Tl [23], Rb, and Ni [32], and other studies which are more focused in food characterization offer some general data for a wider set of minor and trace elements [49,50,51,52] using the inductively coupled plasma techniques for analysis.

Almost all these mineral compounds found in plant tissues are obtained by the plant from the soil, water and fertilizers employed, hence certain variability in the mineral content of almonds is expected to depend on the geographical origin, which combines soil and weather conditions, and agricultural practices [53]. Moreover, another factor that should be borne in mind when interpreting mineral data of almond kernels is the dependence of the product composition on the ripening state [17,54], particularly on Ca and minor elements, such as Zn and Fe. It is important to recall that different almond genotypes could maturate in periods along the year and with different ripening period length [4] and this should be considered when comparing different cultivars although this information is often lacking in the references.

As regards the influence of the cultivar in the mineral content of almond kernels, Drogoudi et al. [33] studied the mineral composition of 72 varieties of almonds produced in three different countries (France, Greece and Italy) harvested in one year or two depending on the chosen variety. According to their results the major elements K, Mg and P, but especially Ca, could be used to mark differences among almond genotypes when one harvesting year was considered. However, Ca variability was high when data were compared using two harvesting years. In the work of Simsek et al. [37], related to the evaluation of the composition of 12 almond cultivars grown in the same year in Southeast Turkey differences were found among cultivars regarding the levels of major elements and Na, Mn, Fe, Cu, and Zn, nevertheless no conclusion was driven regarding the classification of varieties. Following this line, Prats et al. determined the concentration of ten elements in kernels of 19 almond cultivars [55]. The results showed that some of the cultivars from different regions (Americans vs. the Mediterraneans) could be segregated according to their Ca and Fe levels despite having been harvested in the same Spanish geographical area and year. In this line, Ayadi et al. [56] studied the composition of six almond cultivars grown in Tunisia through two harvesting years, with three local cultivars and three originating from other countries. In their study, no significant differences were found for the Mg, P, and K level, however, some differences showed up for the Ca levels among some of the local cultivars and the rest of genotypes. It is interesting to note that in this study rain was the only water source employed in the orchards. In another study, Özcan et al. [57] evaluated the chemical composition of five cultivars grown in the same year in two close provinces of Turkey. No further details of samples were provided, and their results reported differences among the five cultivars mineral composition, although these differences not always were significant. Yada et al. [35] investigated the differences in the composition of seven almond cultivars, included in the top ten almond-producing varieties in California, harvested in the 2005–2007 period in three regions. In this work, authors concluded that although micronutrient profiles obtained for each variety over the three years of the study were notably similar, the variety had a high level of significance for K and Zn concentrations. For these elements, the level of significance for the cultivar effect was higher than for the region and year effects for Zn and higher than for the year effect for K. These results were obtained even each sample was supplied by an independent grower, that is, without control about the orchard management practices. Finally, the work developed by Rabadan et al. [32] investigated the influence on kernel composition of the genotype and weather variables of 10 almond varieties collected at the most appropriate harvest date for each cultivar. In this study, the authors stressed the fact that the evaluation of genotype differences needs to consider the different weather conditions in which the kernels are grown. The obtained results indicate that, concerning the mineral concentration, the variability of the major mineral was lower than that of minor components when dealing with the different cultivar effect, particularly interesting is the fact that K and Mg content variability was mainly explained by the cultivar rather than the harvest year of kernels.

## 7. Vitamin E

Tocopherol content of almonds is important as it protects the fat against oxidation [56]. The main homologue is α-tocopherol with values in the range between 85–840 mg/kg kernel for cultivars from Spain, USA and Italy [58], while γ- and β-tocopherols and α-, β-, γ-, and δ-tocotrienols are presented in meagre quantities [59]. These compounds are usually separated and quantified using HPLC with fluorescence or UV detection [20,60]. Additionally, evaporative light scattering detector (ELSD) can also be used, but it is less sensitive than fluorescence [61].

In the last years, several manuscripts have studied the influence of almond cultivar, harvesting conditions and location of cultivation on the content of antioxidants such as tocopherols. In this way, a review was recently published that compiles the knowledge about this until now [58]. The combination of the year of harvesting, temperatures and location of cultivation seems to have a significant effect on almond fruit development additionally to the type of cultivar. Several studies have demonstrated that, even though the effect of drought stress on tocopherol concentration in almonds is ambiguous, the combined effect of two factors, such as drought and heat, is related to an increase in α-tocopherol content in most of the cultivars. This effect was verified in some studies for almonds cultivated in Morocco, Afghanistan, and Northwestern Argentina [25,29,62]. The year of harvesting also has an important effect on tocopherol content [21]. Furthermore, it must be considered that tocopherol content depends on the almond kernel development state. The time between 95 and 115 days after anthesis was crucial to enhance tocopherol content increasing water and fertilization [5]. Finally, concerning the influence of cultivar in tocopherol content, Kodad and coworkers indicated that α-tocopherol content is under polygenic control which explains the considerable variability among almond cultivars and genotypes [58].

Despite the influence on tocopherol of so many variables, some comparative studies of tocopherol content in almond cultivars from different locations and countries can be found in the literature. In a study in which 20 almond cultivars from Afghanistan were compared, it was found that α-tocopherol content varied in an important range from 139 to 355 mg kg^−1^ in almond kernels [25]. Oher study was centred in the variation in α-, γ-and δ-tocopherol for several samples of the cultivars Butte harvested in California and, Marcona, Guara, and Garrigues cultivated in different locations of Spain during two different years. After applying one-way analysis of variance (ANOVA) to the tocopherol data, it was possible to find significant differences employing only α-tocopherol content between Marcona and the other three cultivars included in the study independently of the year and location of cultivation [9]. Furthermore, some tocopherol homologues content relations could be essential screening markers to study adulterations of almond oils with other vegetable oils, for example, the ratio of α-/ (β + γ)-tocopherols [63].

To characterize almond cultivars chemometric techniques have been applied to tocopherol data. In this line, a study in which the tocopherol composition of 52 almond cultivars grown in the Apulia region (Italy) was carried out [64] (Table 3). Even though significant variability in tocopherol content among cultivars was found, after applying PCA to the data it was possible to classify the Italian cultivars into five groups with decreasing tocopherol content. The group with the highest tocopherol content was composed by SenZarte cultivar with near 800 mg kg^−1^ almond kernel and Rachele cultivar with near 700 mg kg^−1^ and the second group was made up the cultivars Albanese, Zin Zin, Piscalze, and Galgano with tocopherol contents around 500 mg kg^−1^.

However, as tocopherol content alone has limitations for establishing differences among a great number of cultivars, several studies add other components such as fatty acids for the classification. In this way, Maestri et al. found that total oil, oleic acid and total tocopherol could be good markets to find differences among cultivars [62]. So, the four Argentina almond cultivars (Martinelli C. Emilito INTA, Caceres Clara Chica and Javier INTA) presented lower levels of α-tocopherol than typical Spanish ones such as Marcona and Guara or the well-known cultivar Nonpareil. Kodad et al. considered the content of the major fatty acids and tocopherol homologues in 44 almond cultivars originated in different Spanish growing regions. Authors applied a PCA to classify the cultivars included in the study, and it was found that oleic and linoleic acids and δ-tocopherol were important variables for quality characterization of almond cultivars, but they concluded that tocopherol content is not recommended to use as there is high variability in their values among cultivation years [65].

Moreover, the thermal processing of almonds alters the tocopherol profile of the seeds meanwhile the tocotrienols are not affected by the thermal stress. Hence, a 35% decrease in α-tocopherol content was observed when almonds were roasted at 175 °C [66]. In the study it was observed that the higher the temperature reached during roasting; the higher tocopherol losses were noted due to oxidation processes. Similar results were obtained in another study which recorded a 20% decrease in α-tocopherol when almonds were roasted at 140 °C for 25 min and up to 63% when they were roasted at 165 °C for 15 min. Losses in γ-tocopherol were also nearly 20% for both thermal treatments [67].

To sum up, the tocopherol profile is strongly influenced by several parameters not only by the cultivar. This fact conditions the applicability of this parameter alone as a biomarker of almond cultivars improving its applicability when it is combined with other variables such as fatty acids.

## 8. Phytosterols

Phytosterols are recognized for decreasing serum total, low-density lipoproteins (LDL) and cholesterol levels if they are included in the diet regularly in quantities of about 1–2 g per day [68]. There are two main ways of determining phytosterols using GC with FID detector or MS detector. Another possible way is separating the phytosterols together with tocopherols using HPLC with UV detection. An average value of total phytosterol amount in almond kernel ranges from 1100 to 2800 mg kg^−1^ being β-sitosterol the principal component [69].

Rabadan et al. studied the phytosterol composition in ten almond cultivars during two consecutive crop years in the same location by GC-FID [70]. The quantifiable sterols were sitosterol, Δ5,23-stigmastadienol, clerosterol, sitostanol and Δ5,24-stigmastadienol. For these components, it was found that the crop year has a more significant influence on their composition than the genotype. Kodad et al. studied the phytosterol variability excluding steryl glycosides and acylated steryl glycosides in almond germplasm and found that the principal sterol was β-sitosterol (from 55–85%), followed by Δ-avenasterol (8.5–28%) [71]. In this study, it was pointed out the influence of the year of cultivation and the origin. In another work, ten common Californian almond cultivars were compared, and significant variability was encountered with values of β-sitosterol ranging from 103 to 206 mg 100 g^−1^ almond kernel. For stigmasterol, the range of values varied from 1.3 to 9.8 and for campesterol the values oscillated from 4.1 to 11.8 mg 100 g^−1^ almond kernel.

Additionally, free sterols were determined using HPLC-UV with a simple dilution of the almond oil in an organic solvent. Eight known Spanish almond cultivars were analysed and the content of β-sitosterol ranged between 138–249 mg 100 g^−1^ of almond oil [72]. Based on the data published till now total phytosterol composition is highly dependent on environmental conditions [32]. Maybe this is the reason why these compounds have not been employed alone to classify almond cultivars.

## 9. Phenolic Compounds and Antioxidant Activity

The interest in the determination of the antioxidant activity of different almond cultivars has considerably increased in the last years [73,74]. Several assays can be used for almond antioxidant capacity determination, including assays determining the ferric-reducing antioxidant power (FRAP), 1,1-diphenyl-2-picrylhydrazyl (DPPH)-free-radical-scavenging activity, oxygen-radical-absorbance-capacity (ORAC), Trolox-equivalent antioxidant capacity (TEAC) and the method that uses 2,2-azinobis (3-ethyl benzothiazoline)-6-sulfonate (ABTS), among others. Additionally, to identify and quantify the polyphenols present in almond samples, reversed-phase liquid chromatography coupled to mass spectrometry (RP-LC-MS) detection is the analytical technique which provides the best results [75]. In many studies, it has been reported that the use of a single antioxidant method is not adequate considering that different reactive species and mechanisms are involved in oxidative stress in vivo. For this reason, a combination of results obtained by using several of the mentioned methods seems to provide the most reliable tool for the study of almond antioxidant properties [74].

By using several of the mentioned methods, the antioxidant capacity of ethanolic extracts of different parts of the almond fruit such as seed, skin and shell cover were evaluated. The obtained results revealed that the antioxidant capacities of skin and shell cover were significantly higher in comparison with the whole seed at the same extract concentration [73]. In this context, Bottone et al. [76] analysed the antioxidant activity of seeds, skins and blanching water of four Italian almond cultivars (Toritto, Fascinello, Pizzuta, and Romana) by the total phenolic content (TPC), DPPH, and ABTS methods. Although no discrimination was achieved, authors underlined that Toritto almond cultivar showed the highest concentration in phenolic compounds and antioxidant activity.

In a different research work [77], ten almond cultivars were considered (Texas, Jonhson Prolifics, Thompson, Filippo Ceo, Genco, Tuono, Largueta, Marcona, Francolì, and Ferragnès), grown in the same orchard and subjected to the same agronomical regime, to study the effect of cultivar on the nutritional characteristics, in particular, their phenolic composition. Thus, DPPH and TPC were applied showing a wide variability in the phenolic content among almond cultivars ranging from 943.84 for Jonhson Prolifics to 2751.22 mg kg^−1^ gallic acid for Francolì. These results point out the strong influence of the genotype of almonds.

Various studies showed that the flavonoid content and antioxidant activity are more controlled by almond cultivar than by yearly differences [78,79].

Different phenolic compounds were characterized in seed, skin, shell and hull almond extracts in samples of Marcona, Butte, Guara, Planeta, Colony, Carmel, and Padre almond cultivars [71]. The phenolic compounds identified in skin samples allowed finding differences among the cultivars. These results agree with the ones obtained by Garrido et al. [80] carried out in different almond skins related to the phenolic composition. Moreover, in the work carried out by Valdés et al. [72], LDA was successfully applied by using the total phenolic content (TPC), the antioxidant activity measured by FRAP and individual flavonoid contents as predictors, obtaining a 100% correctly classification of the blanched samples according to each cultivar. Similarly, Bolling et al. [79] found that canonical discriminant analysis of polyphenols content and antioxidant activity measured by FRAP could distinguish almonds from different cultivars (Nonpareil, Carmel, Butte, Sonora, Fritz, Mission, and Monterey) harvested in different seasons with 80% confidence. Regarding the TPC expressed as mg gallic acid equivalent (GAE) g^−1^ almond; Butte and Fritz showed the lowest values (58 ± 7) being Sonora samples the ones with the highest value (159 ± 1). In all the samples, the main phenolic compounds were [80,81]: (+)-catechin, (−)-epicatechin, naringenin-7-O-glucoside, kaempferol-3-O-rutinoside, isorhamnetin-3-O-rutinoside, isorhamnetin-3-O-glucoside, and naringenin. The flavonoids isorhamnetin, isorhamnetin-3-O-glucoside, kaempferol, quercetin-3-galactoside, catechin, kaempferol-3-O-glucoside, kaempferol-3-O-galactoside, and quercetin provided the best discrimination between cultivars.

Regarding unblanched raw almond kernels, the antioxidant activity and phenolic profile corresponding to Marcona, Texas and Troito samples grown in Serbia were evaluated by Čolić et al. [18]. The obtained TPC values were 204, 1195, and 271 mg GAE kg^−1^ kernel respectively, and the predominant polyphenol found was catechin, followed by chlorogenic acid and naringenin. These results are in accordance with a previous PCA application carried out by Yildirim et al. that reported the relevance of catechin, caffeic acid, epicatechin, and p-coumaric acid as discriminant parameters to differentiate almond varieties (Cristomorto, D. Largueta, Ferraduel, Ferragnes, Ferrastar, Glorieta, Lauranne, Masbovera, Nonpareil, Picantili, Sonora, Supernova, Texas, Tuono, and Yaltinski) [82].

In other work, the phenolic profile and the total phenols content of Californian samples belonged to Butte, Carmel, Fritz, Mission, Monterey, Nonpareil, Padre, and Price almond cultivars were determined. Note that the skin was the part of the fruit which showed the most distinguished differences among almond varieties taking into account the content of phenolic compounds obtaining values such as 60.2 and 128.6 mg GAE 100 g^−1^ in Fritz and Price, respectively, while the content in the kernels was similar between varieties, within the range of 64.4–70.9 mg GAE 100 g^−1^ [83].

To sum up, the polyphenol profile determined in the whole almond or the skin can be a good biomarker to classify almond cultivars harvested in different years but with the help of multivariate statistical techniques.

## 10. Volatile Compounds

The volatile profile of raw and processed almonds has been extensively studied and a great variety of different chemical compounds have been reported [84]. Regarding raw almonds, some alcohols, alkanes, aldehydes, ketones, and heterocyclic compounds have been described [85] as major components present in the volatile profile. It has been stated that the composition of the aroma is directly related to the almond cultivar [28] and the maturity of the nut [86]. Additionally, different volatile compounds can be produced during thermal processing and storage of the almond samples. Regarding the roasting process, since the Maillard reaction occurs, compounds such as furans, pyrroles and pyrazines are generated. On the other hand, in the frying process compounds like trans, cis-2,4-decadienal and trans, trans-2,4-decadienal are present because of the degradation of the frying oil. Also, high amounts of C6-C9 aldehydes (hexanal, octanal, and nonanal) are generated [87].

To measure the volatile compounds, present in almond samples, gas chromatography-mass spectrometry (GC-MS) coupled with headspace solid-phase microextraction (HS-SPME) [87] is the analytical technique mainly used. By using a new developed HS-SPME method for the extraction and quantification of volatile compounds, Xiao et al. [85] analyzed the volatile compounds present in Butte and Padre almond samples submitted to a dry-roasted process. Because of the roasting process a significant (*p* < 0.05) increase in the number of alcohols, heterocyclic and sulfur-containing compounds and aldehydes was achieved in comparison with raw almond samples. Concerning pyrazines, these compounds were mainly detected in roasted samples since they are by-products of the Maillard reaction, except for 2,5-dimethyl pyrazine, which was also found in raw almonds.

In a study conducted by Beltrán & coworkers, raw almonds from Butte, Guara and Marcona cultivars (*n* = 24) were classified based on the following volatile compounds: nonanoic acid content, nonanal and tetradecanal content quantified by HS-SPME-GC-MS [28]. These volatile compounds were identified as suitable parameters to discriminate among samples belonging to the cultivars Marcona, Guara, and Butte by using LDA as a chemometric tool. In Butte samples, lower amounts of nonanal were obtained as expected due to the lower amount of oleic acid present in this almond cultivar.

In another research work with the purpose to evaluate changes in dark and light-roasted almonds, the volatile profile of samples belonging to Butte and Padre were analyzed over time (6 months) by using HS-SPME [88]. The obtained results showed that the content of some volatile compounds such as hexenal and alcohols like 1-heptanol and 1-octanol changed considerably in roasted almonds over time depending on the degree of roast. Moreover, new compounds, such as ketones and other aldehydes ((E)-2-decenal, 2,4-nonadienal), that were not present in raw samples were identified in the processed ones. In contrast, some compounds decreased in roasted samples (2-methylbutanal, 3-methylbutanal, furfural, 2-phenylacetaldehyde, 2,3-butanedione, 2-methylpyrazine, and 1-methylthio-2-propanol) being the reduction independent of the degree of roast or storage conditions. This trend is similar to other scientific works that presented the amounts of C5-C8 aldehydes as useful predictors of rancidity in roasted almond samples [89,90]. Changes caused by roasting of volatile components of nine cultivars (Amendoao, Molar, Pegarinhos, Bonita, Casanova, Pegarinhos, and Refego, Ferragnès and Glorieta) were monitored by HS-SPME-GC-MS analysis [91]. LDA results obtained from raw samples showed that only seven volatiles had statistical significance (benzyl alcohol, 3-penten-2-ol, guaiacol, benzaldehyde, limonene, 2-heptanol, and 3-methyl-1-pentanol) explaining about 58% of the data that allowed the classification of all cultivars. Considerable changes in the volatile profile were caused by the roasting of almonds. Benzaldehyde, hexanal, phenylethyl alcohol, 4-ethylcyclohexanol, and 6-methyl-5-hepten-2-one were only used to perform a PCA by accounting the 78% of the total variance. As a result, four major groups were separated into the discriminant space.

To study the changes in the volatile profile of almonds derived from the frying process, a study carried out by Valdés et al. by employing HS-SPME confirmed the presence of compounds derived from the degradation of the frying oil such as trans, cis-2,4-decadienal and trans, trans, 2,4-decadienal [87]. Also, higher amounts of hexanal, octanal, and nonanal were obtained as aldehydes derived from the oxidation of the lipid fraction since the analyzed samples were fried almonds submitted to normal and accelerated oxidation conditions.

Regarding the application of the volatile profile compounds as useful parameters to discriminate different almond cultivars under oxidation conditions, oxidized samples of Spanish and American oils were classified correctly according to the cultivar being (E)-2-heptenal and (E)-2-nonenal the variables included in the LDA analysis [92]. Butte cultivar showed higher amounts of (E)-2-heptenal in comparison with Spanish cultivars, as expected since the formation of this compound is related to the decomposition of the hydroperoxides formed from linoleic acid. In consequence, an adequate selection of volatile compounds from almonds can be a good strategy to find differences among cultivars but still, there are no conclusive studies as there is great variability in the extraction process of the volatiles and also in their analysis. Based on this, more efforts must be made in this way.

Bitterness in almonds is control by a single gene. As the sweet allele (Sk) is dominant over the bitter allele (sk) when crossing different cultivars is possible to obtain three genotypes. The homozygous SkSk, which corresponds to a sweet almond, the homozygous bitter sksk and the heterozygous Sksk that can correspond to a sweet or semi-bitter almond. Recently, some volatiles, from the whole profile of almonds, were selected as good biomarkers of sweet heterozygous and homozygous [93]. The most important compound found for differencing the sweet and bitter almonds was the benzaldehyde. Multivariate statistical techniques were applied to the volatile data to know if more accurate classification could be obtained. In this case, not only benzaldehyde, but also benzyl alcohol, 2-methyl propanol, 3-methylbutan-ol, and 3-methyl-2-buten-ol contributed to the correct classification.

## 11. DNA Fingerprinting

OMICS is the suffix employed in some different biological disciplines, such as genomics, proteomics, among others. Genomics is the science that studies the complete structure of the DNA of an organism, all its genes. An important part of the genomics potential is the possibility of identifying certain regions of the DNA organism (i.e., DNA fingerprinting). Those fragments of DNA can be employed as a way of genotypic information [94]. The evolution of this discipline has recently allowed knowing the genome sequence of some almond cultivars such as Lauranne [95] and Texas [96]. This scientific advance opens the possibilities to easily compare almond cultivars and to see the similarities with other Prunus species or for example to find differences among bitter and sweet almonds.

DNA sequencing was first introduced in the 90s using the restriction fragment length polymorphisms (RFLPs) technology. Unfortunately, this method is not nowadays extended due to the complexity and time-consuming of the methodology when the genome sequence is not known. More recently, the polymerase chain reaction (PCR) fragment analysis (DNA fingerprinting) has been commonly cited in the literature for food varietal classification [97]. This methodology allows for the generation of millions of copies of pure DNA sections from a very small sample. Consequently, characteristics DNA sections can be selected as possible biomarkers that let cultivar identification and to find similar genetics among plants. In this sense, some of the most cited methods for amplification of selected sections of the DNA are: (a) random amplified polymorphic DNA markers (RAPD), (b) amplified fragment length polymorphisms (AFLP), (c) simple sequence repeats polymorphisms or microsatellites (SSRs) and more recently, (d) expressed sequence tags (ESTs). According to Martinez Gomez et al., the SSRs and ESTs methods seem to be the best techniques for cultivar identification in Prunus species for the polymorphism of the markers [98]

RAPD are pieces of genomic DNA amplified through PCR using a decamer primer (10 nucleotides long) of random sequence [99]. The methodology was used to study the genetic similarities between 50 accessions of almond cultivars in Australia. A cluster analysis was applied to the data and cultivars originated in Europe and the Middle East were classified in a different group than the almond cultivars originated in California. The origin of some Australian commercial cultivars was inferred by their placement on the dendrogram [100]. In another study, 10 primers were selected using SSRs to study the genetic diversity of Tunisian almond cultivars and their similarities with foreign cultivars. PCA was applied to view the relationships among the 100 almond cultivars included in the study. A strong genetic affinity among cultivars was encountered independently of the geographic locations [101]. Another study employed both RAPD and SSR markers to determine the genetic relationship among almond genotypes from Turkey and other origins. After applying the cluster analysis, a great genetic diversity was found among Turkey almonds cultivars [102].

Other studies have been done to find the genetic relationships from Italian collections [103] and also Iranian cultivars [104]. However, maybe the largest genetic studies were done by the team constituted by Fernandez i Martí and coworkers [105,106]. In a first research, they studied 93 almond genotypes, most of them from Spain and foreign regions. Using 19 SSR markers and after applying cluster analysis, authors managed to associate cultivars based on the genetic closeness [105]. A larger study was conducted by the same group including 158 almond genotypes representatives of the diversity in the five continents. In this work, all samples were compared using 17 SSR markers [106]. As a result, interesting classification from the cluster analysis was obtained. Several groups were initially constituted. The first one was constituted by primitive Iran and Majorca genotypes. The cultivar Texas was classified in this group, and it was explained as it was probably introduced in America by the Spanish missioners. Another group constituted by evolution genotypes from Iran and Mediterranean zones was obtained, and a third group was formed by Californian and Australian cultivars. Finally, there was another group in which all wild species were classified. This study underlined that even though there is a great dispersion among genotypes, they maintained a genetic relation based on their genetic ancestors [106].

A single-nucleotide polymorphism (SNP) is a substitution of a single nucleotide in a specific point in the genome. An SNP-phylogenetic analysis was employed to classify almond cultivars in two main groups [96]. The first association contained the Italian cultivars (Falsa Bares, Geneco and Cristomorto). In the second group, two subgroups were found. One branch was constituted by the US and French cultivars (Ripon, Nonpareil Belle d’Aurons and Ai). The second one was formed by the Spanish cultivars Marcona, Vivot and Desmayo Largueta. The association obtained was following the geographical origin of cultivars.

Genomics has also helped to study the genetic variability of new almond cultivars developed in breeding programs. The real tendency in Mediterranean almond breeding programs is centred in the selections of self-compatibility and late-blooming almonds and the difference between the European and the USA and Australia programs is the soft or hard-shelled preferences. SSR was employed in a recent study to determine the parental relationships of the 220 almond genotypes. The USA developed cultivars showed two main ancestors, Non-Pareil and Mission. Meanwhile, cultivars developed in breeding programs in Spain had three basic clones, i.e., Tuono, Cristomorto, and Primorskyi. Furthermore, the diversity in Australia was not superior with Non-pareil and Lauranne as the main ancestors. Only the Israeli breeding program showed higher diversity with six important ancestors [107]. Experts have pointed out that this limited variability in the breeding lines has conducted to phenotypic depression. Consequently, in future almond breedings programs, inbreeding should be avoided in favour of more genetic diversity [96].

**Table 3:** Selection of some works in which characterization of Almonds of Different Geographical Origins and Cultivars have been done based on the tocopherol, polyphenol and DNA profile.

Geographical Origin	Cultivars	Compounds	Analysis	Ref.
Different cities in Spain and California	Garrigues, Guara, Marcona and Butte	α, (β + γ), δ- tocopherol	HPLC-PDA ANOVA	[9]
Australia, Spain and California	Nonpareil, Johnston, Somerton, Peerless, Price Carmel and Guara	α, (β+γ), δ- tocopherol	HPLC-PDA	[21]
Apuglia (Italy)	Barlettana, Cristomorto, Santoro, Catuccia, Filippo Ceo, Piangente,, Pidocchioso, Tuono, Mincone, Catucedda, Fragiulio, Centopezze, Putignano, Ciavea, Santeramo, Galgano, Irene Lanzolla, Cacciola, Catalini, Rana Gentile, Ferrante, Zin Zin, Trianella, Nocella, Cinquanta Vignali, Pizzutella, Pastanella, Pepparuddo, Aloia, Bares, Pappamucco, Rossa, Reale, Senz’arte, A Grappolo, Albanese, Vuoi o non vuoi, Ficarazza, Giunco di Cozze, Alberobello, Cosimo di Bari, Rana, Primecerio, Lorenza Tribuzio, Piscalze, Antonio De Vito, Monaca, Tondina, Pettolecchia, Pulita, Scorza Verde, Gioia, Rachele.	α-tocopherol	HPLC-PDA PCA-LDA	[64]
5 continents	46 California and Australian, 70 European cultivars from Mediterranean zones, 26 from Iran and wild genotypes.	17 SSR markets	PCR Cluster analysis	[102]
Spain and USA	Marcona, Guara, Planeta, Butte, Colony, Carmel, and Padre	TPC, antioxidant capacity (FRAP), and individual flavonoids content in almond skins	HPLC-ESI-MS/MS LDA	[9]

## 12. Conclusions

The composition of the almonds can be affected by the cultivar and by the environmental conditions and agricultural practices used during their development. After an extensive review of recent literature related to the chemical characterization of almonds, it has not been possible to find a single chemical marker that can be used to unequivocally differentiate different varieties and/or origins of the same variety.

On some occasions, it has been found that the impact of the variables mentioned above makes it difficult to locate these markers, as it has been shown in the case of mineral or protein content. On the other hand, the lack of a unified method of analysis has limited research on the use of parameters such as fibre or carbohydrates as differentiating markers. It is important to mention that the components of the fat fraction have been among the most studied as possible sources of variety or origin markers, although a satisfactory answer has also not been found using a single compound. The most common situation is to find combinations of several components of the fat fraction that have allowed classifications among different cultivars. For this purpose, it is necessary to use multivariate statistical analysis techniques. In this sense, the most promising chemical compounds are fatty acids such as palmitic, oleic and linoleic acids and the oleic/linoleic acid ratio together with the following triglycerides OOO, OLO, POO, OLL, StOO, LLL, and PLL, as well as the -α-/(β+γ)- tocopherols ratio.

The content of flavonoids and certain phenolic compounds such as catechin, caffeic acid, epicatechin and p-coumaric acid could be used as discriminating parameters to differentiate almond varieties since they seem to depend more on the almond cultivar than on other variables. However, these compounds are mainly found in the skin, so their use would not be practical in blanched almonds. In addition, the volatile profile could be a useful tool since some compounds such as nonanal, tetradecanal, and nonanoic acid have shown a good capacity to classify raw or roasted almond cultivars. Nevertheless, the difficulty of the process of extraction and quantification of these volatile compounds limits their application from a practical point of view.

Other methodologies applied to solve this problem are focused on the analysis of the genetic material such as DNA fingerprinting which has proved to be a very powerful technique to identify varieties regardless of the conditions of collection. Another possibility was to differentiate bitter and sweet almond cultivars based on some selected volatiles. The recent advances in genomics suggest that in a near future it could be possible to differentiate almond cultivars based on specific sections of the DNA. Finally, metabolomics can also be a useful tool to classify almond cultivars and the location of the crop. In any case, analysis of the results using multivariate statistical techniques is required.

As a final remark, it should be pointed out that, only on few occasions the methodologies mentioned above have been implemented in routine analysis in the food industry or in official control laboratories since they require expensive equipment, are labour demanding and have low sample yields. In order to solve these problems, some possible alternatives are the analysis of physical properties or even the shape of the almonds but they could not be used in foods in which the almonds had been processed and incorporated into a mixture of ingredients. Therefore, the problem of the classification of almond varieties, from a practical point of view, remains to be solved and further investigation is needed.

## Figures and Tables

**Table 1:** Almond production by top 7 almond-producing countries [2]. Data are expressed in tonnes.

Country	2014	2015	2016	2017	2018	2019
United States	1,545,500	1,302,998	1,376,337	1,476,539	1,872,500	1,936,840
Spain	195,704	211,084	199,167	255,503	339,033	340,420
Australia	55,978	63,331	72,902	75,373	69,880	146,410
Morocco	101,026	97,723	112,681	116,923	117,270	102,185
Iran	136,338	146,000	111,845	129,566	139,029	177,015
Italy	74,016	70,399	74,584	79,599	79,801	77,300
Turkey	73,230	80,000	85,000	90,000	100,000	150,000

**Table 2:** Characterization of almonds of different geographical origins and cultivars based on their fatty acid composition.

Geographical Origin Confirm the Color of Back Ground	Cultivars-Country of Origin	Compounds	Analysis	Ref.
Turkey	Cristomorto, Largueta, Ferraduel, Ferragnes, Ferrastar, Glorieta, Lauranne, Masbovera, Nonpareil, Picantili, Sonora, Supernova, Texas, Tuono, and Yaltinski	Palmitic, Palmitoleic, Heptadecanoic, Stearic, Oleic, Linoleic, Arachidic	GC-FID PCA	[7]
Sicily, Spain, California	Not specified	Oleic, Linoleic, Palmitic, Stearic, Myristic, Arachidonic, Arachidic	GC-MS PCA	[17]
Serbia	Marcona-Spain, Texas- USA, Troito-Italy and 17 selections from the large spontaneous population of almonds in North Serbia, called Slankamen Hill	Oleic, Linoleic, Palmitic, Stearic, Myristic, Arachidic, Palmitoleic, Heptadecanoic, Cis-10-heptadecenoic, Linolenic, Eicosenoic, Tricosanoic, Behenic, Pentadecanoic, Docosadienoic, Lignoceric	GC-FID PCA	[18]
Afghanistan	Khairodini samangani, Pista Badam, Kaghazai Siah Dana, Qaharbai, Sangak Shashum, Shokorbai, Carmel, Kaf Samangani, Khairodini, Kaghazai Kalan, Sattarbai, Belabai, Marawaja Du Maghza, Sattarbai Doum, Shakh-i- Buz Safid, Sangak Dahum, Qambari Kunduzi, Sattarbai Bakhmali, Khairodini-161 Samangan, Sattarbai Saiz Talkhak	Palmitic, Tridecanoic, Palmitoleic, Stearic, Oleic, Linoleic, Arachidic, Linolenic, Henicosanoic, Behenic, Tricosanoic, Lignoceric	GC-FID PCA	[24]
Argentina, France, Greece, India, Italy, Portugal, Spain, Syria, Ukraine, USA	Emilito-Argentina; Marcona Argentina-Argentina, Ai-France, Ardechoise-France, Bartre-France, Belle d’Aurons-France, Cristar-France, Ferragnes-France, Ferralise-France, Fourcouronne-France, Fournat de Brezenaud-France, Pointu d’Aureille-France, Princesse-France, Stelliette-France, Tardive de la Verdiere-France, Tournefort-France, Exinograd-Greece, Phyllis-Greece, Pagrati-Greece, Symmetriki-Greece, Truoito-Greece, Tsotoliou-Greece, Kata-India, Spilo-India, Talengy-India, Bonifacio-Italy, Cavaliera-Italy, Cristomorto-Italy, Filippo Ceo-Italy, Fiori-Italy, Fragiulio-Italy, Mollese-Italy, Olla-Italy, Prouvista-Italy, Rachele-Italy, Rana-Italy, Supernova-Italy, Tuono-Italy, Carreirinha-Portugal, Cosa Nova-Portugal, Gama-Portugal, Rameira-Portugal, Raposa-Portugal, Verdeal-Portugal, Atocha-Spain, Del Cid-Spain, Desmayo Largueta-Spain, Desmayo Rojo-Spain, Garbí-Spain, Garrigues-Spain, Mollar Arbeca-Spain, Marcona-Spain, Mollar-Spain, Ramillete-Spain, Verdereta-Spain, Siria-1-Syria, Siria-3-Syria, Nikitskij-Ukraine, Primorskij-Ukraine, Sovietskij-Ukraine, Drake-USA, IXL-USA, LeGrand-USA, Mono-USA, Nec Plus Ultra-USA, Nonpareil-USA, Peerless-USA, Tardy Nonpareil-USA, Texas-USA, Thompsom-USA, Tioga-USA, Tokyo-USA, Yosemite-USA	Palmitic, Palmitoleic, Stearic, Oleic, Linoleic	GC-FID PCA	[25]
Portugal	Protected Designation of Origin: Casa Nova, Duro Italiano, Pegarinhos, Refego. Non PDO: Ferraduel, Ferragnes, Ferrastar, Gloriette and Marcona	Palmitic, Palmitoleic, Cis-10-heptadecenoic, Stearic, Oleic, Linoleic, Arachidic	GC-FID PCA	[27]
Spain and California	Marcona-Spain, Guara-Spain Garrigues-Spain and Butte-California	Palmitic, Palmitoleic, Stearic, Oleic, Linoleic	GC-FID PCA	[28]
Morocco; Spain, France and Tunisia	Marcona-Spain, Desmayo Largueta-Spain, Ferragnès-France, Fournat de Brézenaud-France, Ferraduel-France, Khoukhi’-Tunisia and 46 local genotypes from the Rif mountains (north of Morocco), the Atlas mountains and the valley of Tadla (central-south Morocco)	Palmitic, Palmitoleic, Stearic, Oleic, Linoleic	GC-FID PCA	[29]

GC-FID: gas chromatography-flame ionization detector; PCA: principal component analysis; GC-MS: gas chromatography-mass spectrometry.

## Data Availability

Not applicable.
